# Transformational leadership in development of transformative education in nursing: a qualitative study

**DOI:** 10.1186/s12912-022-01154-z

**Published:** 2023-01-13

**Authors:** Azam Ghorbani, Nooredin Mohammadi, Zahra Rooddehghan, Fatemeh Bakhshi, Alireza Nikbakht Nasrabadi

**Affiliations:** 1 Medical Surgical Nursing Department, School of Nursing and Midwifery, Tehran University of Medical Sciences, Tohid Square, Tehran, 1419733171 Iran; 2 Nursing Care Research Center, School of Nursing and Midwifery, Iran University of Medical Sciences, Rashid Yasemi St, Valiasr St, Tehran, 1996713883 Iran; 3 School of Nursing and Midwifery, Tehran University of Medical Sciences, Tohid Square, Tehran, 1419733171 Iran; 4 Research Center for Nursing and Midwifery Care, Shahid Sadoughi University of Medical Sciences, Yazd, Iran Safaeih, Buali St., Yazd, 8916877443 Iran; 5 School of Nursing and Midwifery, Tehran University of Medical Sciences, Tohid Square, Tehran, 1419733171 Iran

**Keywords:** Leadership, Nursing Education, Qualitative Research, Higher Education

## Abstract

**Background:**

Regarding the dynamic and increasing needs of communities, changes in the education system are essential to train competent healthcare professionals. The study aimed to explore the experience of educational directors, teachers, and students to gain insight into the implementation of educational transformative programs.

**Method:**

A qualitative approach with the grounded theory method was applied in this study. Twenty-four participants were selected by using a purposive and theoretical sampling method. The data were collected from April 2019 to May 2020 in nursing schools of Tehran, through in-depth semi-structured individual face-to-face interviews and field notes. Collected data were analyzed by Corbin and Strauss’s (2015) approach.

**Results:**

In this study, transformational leadership was extracted as the core concept. The core concept emerged from four sub-concepts including transformative management; educational policy requirements; providing a platform and community-centered education.

**Conclusions:**

Nurse educational directors need to achieve some competencies and capabilities for implementing transformative education in nursing schools. Also, achieving a transformative perspective by educational directors is essential.

## Background

Transformation in the healthcare education system is inevitable which is required to make a balance between the healthcare educational system and community demands [1, 2]. Several leading factors trigger educational organizations to begin transformational changes and reform the education system [3]. The main factors include ever-changing needs in the community, rapid advances in medical science, globalization, commercialization of higher education, and incompatibility between healthcare professionals’ qualifications and their competencies [1, 2].

In addition, advanced new and emerging technologies are acknowledged as influential factors in the transformation of the education system [1, 4]. The new generation of learners prefers technology-based communication. They expect their education to be integrated with technology and have become accustomed to high-tech learning environments [5]. Also, the evolution of psychological and educational theories has resulted in the development of human-centered learning methods [6]. During recent decades, education systems experienced major reforms related to the development of psychological and teaching–learning theories and the advancement of educational technology [2].

Accordingly, reforming the nursing education systems is required to address the development of the teaching–learning process and ever-changing healthcare needs in the communities [7, 8]. Nurses play a key role in the health system. As a result, nurse educators must implement the required measures to transform and improve nursing education systems [9] and prepare students to deal with today’s complex and evolving healthcare settings [1, 10, 11].

Transformative education (TE) is a recommended strategy to improve the quality of education and health systems [1, 11]. TE is an approach that claims to change the common concept of education which merely focus on transferring knowledge from the teacher to the learner [12]. The application of TE in higher education has led to knowledge construction, and ontological and epistemological transformation [13]. TE seeks to develop processes that result in long-lasting learning changes rather than immediate and dramatic change [14].

The TE approach is launched through a set of educational reforms such as adopting competency-based approaches, developing interprofessional education, promoting teamwork, and developing the teaching capabilities of faculty members [1]. According to Renigere [15], TE leads to the development of nursing education consistent with the global trend for transformation in education and learning [15]. Tsimane and Downing [16] mentioned that nursing students need to participate in self-directed learning and reflection. Teachers as well are required to move towards transformative changes by reducing traditional educational methods and strategies [16]. Bvumbwe and Mtshali [17] said transformation in nursing education is needed more than ever. If nurses want to act effectively and have a positive impact on patient outcomes and care, nursing education must be consistent with global changes and challenges [17].

As a need for today’s education, TE aims to empower learners to play an active role in all areas of social and professional life [18]. TE aims to train workforces who are qualified and apply global knowledge to provide context-based and international-oriented services. These workforces are competent to respond to the growing and ever-changing needs of the people [1]. In this regard, in Iran, the higher health education system recently has been launching a reform plan in the healthcare education system. This transformational program aimed to provide a comprehensive reform plan. The most important dimensions of this plan include foresight, moving towards third-generation universities to create social welfare, responsiveness, and justice-oriented education, promotion of professional ethics, the internationalization of medical science education, and the development of virtual education. Some new policies of this plan include the institutionalization of an accountable education approach in the health system, developing of equity in medical education, developing of technology-enhanced learning, networking in the medical education system, the institutionalization of professional ethics, improvement the workforce in higher education of health, and decentralization in the health education system [19].

Some elements of TE that are mentioned in these packages are the determination of the priorities of medical education based on national needs, the formulation of codes of practice, and instructions for the delegation of authority to the mega-regions. Before the division of medical sciences universities across Iran into 10 major regions, the power and governance were concentrated with the ministry of health. The creation of mega-regions was done for decentralization and to reduce the need for students to migrate to other cities for medical education. This project aims to develop medical science education, the organization of educational hospitals, and the development of medical science assessments [19, 20]. At the university and school level, these packages and general policies lead to more detailed and practical programs such as the virtualization of exams or holding some virtual courses in the school, holding joint international university courses and scholarships for students, and accreditation programs [19].

TE requires the presence of transformational leaders. Leadership activities in educational processes affect the learning-teaching quality and educational outcomes [21, 22]. Educational leaders have remarkable power to succeed in learners’ learning outcomes and educational reforms [23]. Literature emphasized that leadership behaviors and performance greatly impact the function of organizations and their outcomes [24, 25]. Transformational leaders influence their employees by creating values such as honesty, loyalty, and fairness and emphasizing values such as justice, equality, and human rights and creating organizational changes [26]. Transformational leaders motivate their employees to cooperate and self-leadership with their charismatic personalities, behavior, and relationships. These leaders have an inherent motivation to sacrifice and consider the benefit of the larger society and the organization [27].

Based on the above-mentioned, this qualitative study explored the experience of educational directors, teachers, and students in terms of the implementation of educational transformative programs. The purpose of this study was to determine the role of transformational leadership in the formation of TE, as well as what approaches and strategies leaders use in the formation of TE to the ground and create a platform for the type of education and to face its obstacles and problems.

## Methods

### Study design

This is a qualitative study with a grounded theory approach that explored the process of transformative education formation. The study is directed by the symbolic interactionism viewpoint. The educational processes require interactions between the student and the teacher, the teacher and the educational directors, and the directors with each other. Symbolic interaction regards an individual’s perception of the world around them is created through interactions [28]. In symbolic interactionism, people play an active role and share their attitudes and responses to specific situations with group members and act according to their interpretation of that situation [29]. In the current research, our main assumption is in line with the symbolic theory. We tried to understand the process of formation of transformational education, the views of the participants, their interactions, their interpretation of TE, challenges, and problems, the strategies they choose, and the use cases of those strategies. Considering that the education process is interactive and a social phenomenon, therefore, grounded theory is a suitable research approach for this study. Grounded theory is a research approach that studies human phenomena in the context of social interactions as experienced by people [30]. Also, the Corbin and Strauss 2015 approach was used [31]. This study was conducted from April 2019 to May 2020 in nursing schools in Tehran. We followed the consolidated criteria for reporting qualitative research standards to report this study [32].

### Participants and data collection

Study participants included the educational directors (vice-chancellors and deans), teachers, and nursing students (studying for an undergraduate bachelor’s degree, postgraduate master, and doctoral degree). Directors who conducted the programs for the above-mentioned reform and transformational packages at the university and the faculty, as well as teachers and students who participated in the programs were interviewed. The inclusion criteria were as follows: for educational directors, the experience with designing and implementing an educational transformative program; and regarding teachers or students, the experience with attending an educational transformative program or applying a new educational approach (such as virtual education). Initially, participants were recruited by purposive sampling. Then we used theoretical sampling to advance in-depth data gathering [31, 33].

We conducted data collection using face-to-face semi-structured interviews, field-notes taking, and taking memos. The first author conducted one-on-one interviews using an interview guide. It was developed based on the study objectives. The interviews commenced by giving a brief introduction about the study to the participants. Then they signed a consent form. We informed participants that their partake is voluntary and that the data will be treated securely. They were assured have the option of refusing to answer any question and terminating participation at any time.

The main interview questions included: Describe your experience regarding the design and implementation of an educational transformative program. What facilitators and contributors applied? What challenges and barriers did you face? What strategies did you take to overcome them? Can you explain your experiences regarding attendance in a new teaching program (such as applying a new teaching method or changing the current program)? Have you used new educational approaches in your curriculum? Describe your experience. Then, probing and exploratory questions were asked to encourage further in-depth description and reflection [34]. The interview took place based on the interviewees’ compliance. Each interview lasted approximately one hour on average, ranging from 45 to 90 min. All interviews were audio-recoded and transcribed professionally. The first author recorded field notes and memos concurrent with data gathering. To prevent misinterpretation of the data and to complete their theoretical richness, the researcher started writing notes in the field immediately after each interview. Simultaneously with the analysis of the interview, the field notes were also analyzed.

### Data analysis

Data were analyzed by the Corbin and Strauss 2015 approach [31]. This approach directs by the steps including identifying concepts, developing concepts, analyzing data for context, bringing the process into the analysis, and integrating categories [31]. MAXQDA10 was used to manage the data and to facilitate data analysis.

The process of achieving over 90 percent inter-rater reliability was accomplished in two steps: first, two first authors participated in coding five initial interviews independently and simultaneously. Then they met to compare coding results to achieve consensus and calculated the percentage of the agreement. Second, two coders continued the coding process independently and calculated the percentage of agreement by discussion in the meeting. The entire coding was consolidated through reviewing transcripts, codes, and emerging themes by the third author. The three first authors met to discuss comparing and completing code and themes and triangulate our perspectives. Eventually, all authors assembled to provide feedback on the results of the analysis.

Data collection, coding process, and constant comparison analysis continued until theoretical saturation was observed and no new insights were gained [31, 33].

### Rigour and trustworthiness

Considerations of rigor and trustworthiness were addressed by adopting Guba and Lincoln’s (1985) guidelines [35]. The credibility of our study is demonstrated by providing a thick description of the study protocol, theoretically sampling for a variety of clients’ perspectives, and using direct quotes from participants through the presentation of findings. Member-checking occurred by sharing and discussing findings with participants. They confirmed that the data were representative of their experience. The acceptable inter-rater agreement between coders supported peer-checking. Multiple researchers with experience in qualitative research methods in the research team reflected on the analyzed data to meet researcher triangulation requirements. We considered transferability by providing a rich description of data collection, analysis processes, and findings. By this method, we enhanced the applicability and generalizability of results in other similar contexts.

### Ethical consideration

The project was approved by the institutional review board of the Tehran University of Medical Sciences (coded IR.TUMS.VCR.REC.1397.811).

## Results

Participants included 24 individuals (13 females and 11 males) of which four had educational director positions, 11 were teachers (faculty members) and nine were students in different educational levels. The average teaching and management experience were 13.87 ± 9.09 and 10.33 ± 6.80 years, respectively (Table 1).

**Table 1:** The information of the participants

Participant Code	position	Years of Teaching Experience (years)	Years of Executive Experience (years)	Level of Study	Semester (of studies)
D1	Educational Director	21	10	-	-
D2	Educational Director	31	15	-	-
D3	Educational Director	26	20	-	-
D4	Educational Director	11	11	-	-
T1	Teacher	4	-	-	-
T2	Teacher	25	5	-	-
T3	Teacher	20	-	-	-
T4	Teacher	20	1	-	-
T5	Teacher	7	-	-	-
T6	Teacher	5	-	-	-
T7	Teacher	7	-	-	-
T8	Teacher	11	-	-	-
T9	Teacher	7	-	-	-
T10	Teacher	10	-	-	-
T11	Teacher	3	-	-	-
S1	Student	-	-	Ph.D. Candidate	7th
S2	Student	-	-	Ph.D. Candidate	7th
S3	Student	-	-	Ph.D. Candidate	4rd
S4	Student	-	-	B.Sc. Student	4rd
S5	Student	-	-	B.Sc. Student	5rd
S6	Student	-	-	B.Sc. Student	5rd
S7	Student	-	-	B.Sc. Student	4rd
S8	Student	-	-	M.Sc. Student	4rd
S9	Student	-	-	M.Sc. Student	4rd

Transformational leadership was extracted as the core category in this grounded theory and appeared as a core variable in the process of formation of TE. The main concern expressed by the participants was the difficulty of adapting and aligning with TE. It was related to various factors such as resistance of teachers and students, common use of traditional learning methods, lack of requirements for innovative education, limitation in empowerment, and educational policy-making deficiencies. Transformational leadership in the educational system, by applying the following strategies and approaches tries to eliminate challenges face by TE formation: transformative management; requirements on educational policymaking; providing a platform to transform education, and community-centered education.

- Transformative management: to be transformational, the system first needs transformative managers. Management is the art and technique of effective and efficient use of material and human resources in planning, organizing, and guiding the organization to achieve its goals. The personal and professional performance of managers plays an important role in solving challenges and problems and achieving the goals of the organization.

“We need to optimistically manage conflicts. My motto was that the voice of the opposition should be heard. We can ask opponents to share their alternative views and plans…” (Educational director 3)

Participants emphasized positive attitude and belief toward change and being responsive as important factors to be a change agent manager to initiate innovative actions in the system.

“If university directors don’t carry the attitude to change, they don’t mind about its necessity for our system, or don’t take it into consideration as an opportunity, there is no obvious outcome!” (Educational director 2)

Transformative managers must have managerial characteristics and capabilities. Participants mentioned a wide range of competencies, including high emotional intelligence; being a forerunner, realistic, unidealistic, and energetic; having the courage for change, genius to discover and create opportunities. Based on the results of the study, other managerial abilities of transformative managers were situation analysis, conflict management, spending energy and time, seeking advice, and participatory management. Managers encourage their employees to be creative in solving problems. They motivate them to look at problems from different perspectives and implement innovative problem-solving techniques.

“We must act as change agents and accept challenges. We shouldn’t suppress disagreements, we need to optimistically manage conflicts, and benefit from their advantages. My motto was that the voice of the opposition should be heard. We can ask opponents to share their alternative views and plans… what they would do if they were in the administrative position?!” (Educational director 3)

“Maybe it was the belief in transformations that guided me. I believed that there should be a change in our system, the grades were not real! Some teachers are committed to grading, while others are not! (Educational director 4)”

“One of the main challenges in the way of implementing new educational programs and creating transformation is the lack of support and resistance of human resources. To overcome it, we need managers with a high political ability (Educational director 1)”

- Requirements on educational policymaking: The next element to become a transformative leader and change the system is the quality of educational policies. Policymaking is of great importance and guides the functioning of education systems. Optimal educational policies are those which are scientific, accurate, futuristic, explicit, and accepted by their target community. Participants believed that the prerequisite toward innovative educational approaches is the alignment of policies with these programs. As a result, it will be supported by financial resources and appropriate context to apply innovative approaches.

“See, policies are principal! The system needs to develop active learning methods! in what way the budget is allocated for this field? how well policies are developed? to what degree it is included in the accreditations? I think it’s essential. The more attention innovative learning methods receive, the more teachers will appreciate its significance in teaching…” (Teacher 7).

"Transformative programs must be based on needs and be formed in response to needs. We experienced a period of war in our country, and we tried to answer the need of that time… but from that time, we had to be forward-thinking. We were looking to develop higher education levels as well. We couldn’t stop just because we’re in wartime " (Educational director 2)

Another issue mentioned by the participants regarding the implementation of transformative programs was the necessity of planned and step-by-step actions to apply for new programs in the system. It takes time for revealing the explicit benefits of the change and attracting individuals’ participation.

“At first the program commenced, we faced with a lot of resistance… the time passed, and the teachers and the students become adapted… Its benefits became obvious. Its goal was to make a difference and individuals broke traditional manners!” (Educational director 4).

An example of a field note: "In the meeting of the internal surgery group that was held on the date of … at 11:30, changes were made in the educational program of the master's students. In the new program, these students are assigned to instruct nursing internships. In this program, according to the policy change, facilities are also considered, including the use of a skill lab for practice, the use of guidances, and the use of motivational factors to motivate master students, such as presenting a teaching certificate.

- Providing a platform to transform education: Transformative leaders need the appropriate context and facilities to implement transformative programs. Necessary and important factors in the development and advancement of organizational goals are available resources, which include the workforce, financial support, physical resources, and infrastructures. One of the main characteristics of transformational leaders and managers is the ability to manage and use resources correctly to achieve the goals of the organization. Participants expressed that the provision of an appropriate structure, space, and adequate workforce, the allocation of sufficient time, and financial support as requirements for the implementation of innovative educational programs.

“…normally, any new and transformational action requires time-consuming substructures-building, need to expend considerable efforts to make this happen, and spending a lot of initial costs.” (Educational director 1)

Another participant stated:

“We are designing our educational programs by developed ones in the world, but they need facilities, and an experienced workforce to be implemented…” (Educational director 2)

“The choice of the teaching method is largely not in my control, for example, my classroom has not a speaker! How it’s possible to show an educational video?! Or, for example, the video projector did not play the colors well, this greatly reduces the quality of the work!” (Teacher 4)

With the rapid development of science and technology, the issues related to science and technology have become more complex, diverse, and comprehensive. So, Interdisciplinary communication can be an effective way to exchange the knowledge and information needed. Another factor mentioned by the participants regarding preparing the platform for moving toward educational developments was the need to establish interdisciplinary interactions.

“Innovation in education requires interdisciplinary communication. For example, creating virtual content is completely interdisciplinary… there must be a person who is expert in content production… technical engineers who design and provide the software requirements… and educational program designers who design in what way we teach the content to achieve effective learning” (Educational director 1).

“As a rule, because the generation has changed, their interests have changed, most of the students have become software-oriented, they are very fond of applications.” (Teacher 1)

- Community-centered education: The requisites of transformational leadership are considering the characteristics, conditions, and needs of the community for which programs are designed. For the success of any program, acceptance, and adaptation of the target community with that program is essential. This requires developing programs based on the community’s needs.

“A transformative program must respond to the needs of the present and the future. The needs that exist in our system now, and the needs that may arise in the future.” (Educational director 2)

Another participant expressed:

“The student in the class seem to avoid listening! But they like to play with their phones! I think that sending an online movie to them might be useful… Can I engage them that way? We deal with an internet-oriented generation… well, we must use more virtual-based teaching methods!” (Teacher 1)

**Fig. 1:**
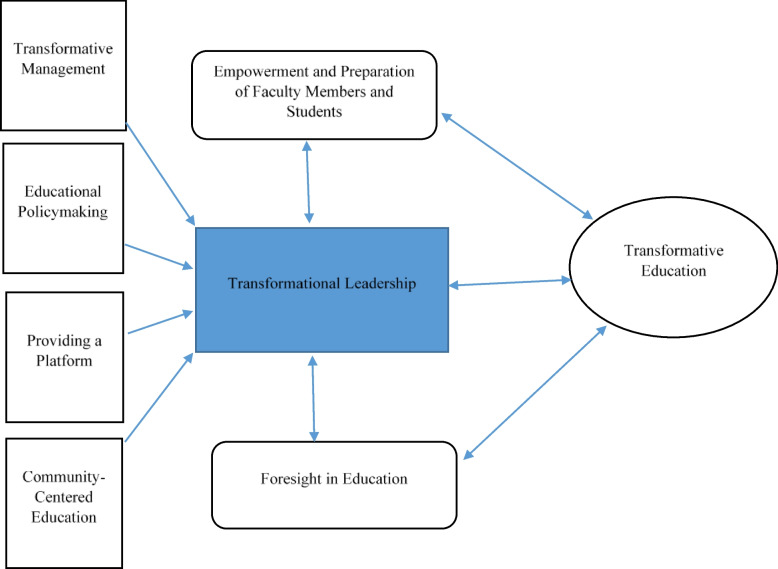
The theory of transformational leadership in formation of transformative education

Based on the analyzed data using the grounded theory approach, we depicted the theory of the formation of transformative education (Fig. 1). Transformative education is a type of education that is forming in the educational system. Nursing education is also moving from a traditional and passive educational system to a transformative educational system. In this regard, students, teachers, and directors as stakeholders of the educational system feel difficulty to adapt with a transformative education. It is related to factors such as the common use of traditional methods, lack of innovative education requirements, and weakness in educational policy. To eliminate the challenging factors to adapt to transformative education and move toward it, the educational system uses strategies and approaches.

One of these strategies is transformational leadership which provides the necessary context and facilities for transformation by transformative management and policymaking in the educational system. Empowerment and preparation of faculty members and students who are the major stakeholder of this education are necessary to achieve effective learning, which is one of the main goals of transformative education. Anticipating and being prepared for unexpected issues which the education system will face in the future are other strategies.

## Discussion

This study was conducted to determine the role of transformational leadership in the formation of TE in nursing education. In this study, the strategies and approaches that transformational leaders need for the formation of TE and facing its obstacles and problems were determined. According to the results, transformational leadership is a core variable in the process of the formation of transformative education. In educational institutions, leaders play an important role to achieve educational outcomes [21, 22]. Leaders who apply a transformational leadership approach, motivate staff and learners [36], improve educational outcomes, and minimize concerns regarding learning outcomes [37]. To form a TE, transformational leaders use four strategies including transformational management strategies, educational policy requirements, providing a platform for educational transformation, and community-centered education. Transformational leadership is the cornerstone and main strategy for the formation of TE. This strategy manages the education system and provides the necessary facilities for transformation and overcoming problems and obstacles by considering the needs and demands of society.

The requisite for transformational leadership is transformative management. For transformationalism, the system initially requires transformative managers. They are concerned agents of change with managerial knowledge and skills. According to Puaca [38], leadership includes a set of professional activities and the competencies of managers are the core activity of the organization [38]. Eddy [39] stated, leadership competencies include a set of knowledge, skills, and abilities of a leader, which leads to the use of various methods by the leader to achieve the goals of the organization [39]. Therefore, leaders must be people-oriented, process-oriented, thoughtful, and correspondent leaders’ support and contribute to change [24, 25, 40]. Transformational leaders influence their employees and subordinates with inspiring power and are the starting point of transformation and change. Concerned with progress and improvement, they push their organization forward, create motivation, support change, and seek creative and innovative ideas.

Educational policies are the second most important element for transformational leadership in the system. If educational policies have a positive approach to change and support new programs and ideas, they create opportunities for change and progress for the system. These policies must be forward-looking, and progressive. Based on Sahu et al. [41], one of the roles and responsibilities of educational administrators is to provide a correct definition of the vision, mission, and policies of the institution [41]. According to Gambhir et al. [42], the policies and procedures of the organization affect the quality of education. The extent to which goals are achieved depends on how well the director’s competencies and self-evaluation policies are supervised [42] The lack of a specific policy in the field of the health system for the development of human resources has created inconsistencies and is considered a threat to the health system to achieve its goals [19]. Therefore, incorrect policy-making without foresight and research is the main obstacle against the currents of transformation, which can divert the orientation of transformation.

The next element for the transformational leadership of the educational system is to provide a platform to transform education. Transformational policies and programs will be implemented in case the appropriate context is prepared. Requirements for implementing transformative education included: adequate financial resources and support; creating a suitable educational structure and space; providing the necessary facilities and resources; and providing a sufficient number of teachers, clinical instructors, and a skilled teaching workforce. Gambhir, et al. [42] emphasized facilities, teachers and staff, financial resources, and financial stability as factors that affect the quality of education [42]. Various factors, including educational policies, budgets, regulatory mechanisms, etc., are effective in the quality of trained human resources in the health system [43]. Therefore, educational leaders must have the necessary competencies to plan and implement programs and manage the human, physical and financial resources of institutions [39, 44]. Physical resources and educational facilities are effective factors in the quality of education, which are positively related to the learning outcomes of learners [42]. In the present study, the participants considered the lack of infrastructure and suitable educational facilities as obstacles to using the method. According to the study of Keshavarzi et al. [45], the lack of infrastructure and required technology was one of the obstacles to using virtual education [45].

The final extracted element was community-centered education. The findings of the study showed that educational programs must be community-based to become administered and accepted by stakeholders. Moreover, the psychological needs, values, and beliefs of society must be taken into consideration. Paying attention to the needs of the target community is effective even in choosing educational tools, including digital tools, and they must be adapted to the needs of the profession and educational program [46]. Literature show that context is one of the influential factors in the performance of leaders and the effectiveness of their leadership. Context affects not only the performance of leaders but also how leadership activities and efforts are coordinated [24, 40]. Lai [47] stated that the atmosphere and culture of the institute is an effective factor influencing the adoption of leadership approaches in educational institutions [47]. Srisaen’s [48] study showed that differences in organizations affect leadership performance and different leaders adopt various approaches and strategies to meet different organizational needs [48]. The organization needs to be properly prepared for the process of transformation. The requirement is the existence of a suitable organizational culture that supports the change and the presence of managers who are concerned about change and transformation, and who prepare the organization’s atmosphere for these types of programs and transformations.

### Limitations

One of the limitations of the study was the limited transformational and innovative policies and programs in the current educational program. This led to limited accessible and eligible participants. However, purposive sampling was used to reach informed participants and data saturation was achieved, and maximum sampling variation was done. To access the informed participants, the researcher contacted educational directives responsible for the transformation and innovation plan in the university, and teachers who were known for using novel educational methods, and students who participated in the classes of innovative professors. Also, each participant was asked to introduce appropriate other ones. Another limitation of the study, which exists in all qualitative studies, is the limitation in the generalizability of the study. To address this limitation, we provided a full explanation of the data collection and analysis process, the characteristics of the participants, and the context.

### Suggestions for further research

Due to the limited study related to TE and its elements, further studies in other contexts and diverse educational communities and systems are recommended. The obtained model and theory should be implemented as a pilot in one of the nursing schools. Also, studies to review the proposed theory and model and the concepts obtained from this study in other contexts and countries are recommended. It is also suggested to explain the lived experiences of managers and educational policy makers in the implementation of transformative programs. This study was conducted in the context of nursing education. We may benefit from similar studies in other fields of medical and basic sciences.

## Conclusion

Transformative education is a requirement for modern education to achieve qualified graduates and increase the efficiency and quality of the education system. According to the study findings, transformational leadership is one of the most important components for shaping the process of transformative education and the core variable. Transformational leadership is the main strategy for the formation of transformational education, which provides the necessary context and facilities for transformation through appropriate management and policymaking of the educational system. This leadership approach seeks to develop the system and staff, achieve goals, and improve educational outcomes. One of the important factors to move towards TE is the training and preparation of transformational leaders in the education system, who have a vision and attitude toward transformation and have the necessary knowledge and qualifications to guide the educational system and manage resources. Moreover, with the power of inspiration, these leaders can create the necessary motivation in employees and colleagues to achieve goals and improve educational outcomes. In addition, foresight and getting ready to manage the issues facing the educational system are also important elements for the formation of TE.

## Data Availability

The datasets generated and/or analyzed during the current study are not publicly available due some interviews contain information that reveals the identity of individuals but are available from the corresponding author upon reasonable request.

## References

[1] Frenk J, Chen L, Bhutta ZA, Cohen J, Crisp N, Evans T (2010). Health professionals for a new century: transforming education to strengthen health systems in an interdependent world. Lancet.

[2] Han ER, Yeo S, Kim MJ, Lee YH, Park KH, Roh H (2019). Medical education trends for future physicians in the era of advanced technology and artificial intelligence: an integrative review. BMC Med Educ.

[3] Hakak M, Hozni SA, Shah SN (2018). Third Generation University is an indispensable necessity for health education. J Med Educ Dev.

[4] Nekuzad N, Sobhani A (2015). Data collectionon the formation of the structure of medical education development centers in Iran. J Educ Stud Nama.

[5] Guze PA (2015). Using technology to meet the challenges of medical education. Trans Am Clin Climatol Assoc.

[6] Lytras M, Sarirete A, Damiani E (2020). Technology-enhanced learning research in higher education: a transformative education primer. Comput Hum Behav.

[7] Capudi LJ (2017). Innovation in nursing education revisited. Nurs Educ Perspect.

[8] National League for Nursing (2005). Transforming nursing education. Nurs Educ Perspect.

[9] Benner P (2012). Educating nurses: a call for radical transformation—how far have we come?. J Nurs Educ.

[10] De Santis P, Willis O (2016). From Karamzin to Putin: transformative learning in practice. Int J Arts Sci.

[11] Franco da Rocha Tonhom S, Guimarães da Costa MC, Galli Hamamoto C, Maria Francisco A, Maria Moreira H, Gomes R. Competency-based training in nursing:Limits and possibilities. Rev Esc Enferm USP. 2014;48(Esp2):213–20. 10.1590/S0080-623420140000800031 25830758

[12] Yacek DW. Should education be transformative?. J Moral Educ. 2020;49(2):257-74. 10.1080/03057240.2019.1589434.

[13] Gal A, Gan D (2020). Transformative sustainability education in higher education: activating environmental understanding and active citizenship among professional studies learners. J Transform Educ.

[14] Hoggan C, Kloubert T (2020). Transformative learning in theory and practice. Adult Educ Q.

[15] Renigere R (2014). Transformative learning in the discipline of nursing. Am J Educ Res.

[16] Tsimane TA, Downing C (2020). Transformative learning in nursing education: a concept analysis. Int J Nurs Sci.

[17] Bvumbwe T, Mtshali NG (2018). Transforming nursing education to strengthen health system in Malawi: an exploratory study. The Open Nursing Journal.

[18] Ghorbani A, Mohammadi N, Rooddehghan Z, Bakhshi F, Nikbakht NA (2022). Effective factors and challenges of forming transformational education in the nursing education system: a qualitative study. Int J Nurs Educ Scholarsh.

[19] Pourabbasi A, Akbari H, Akhvan AA, Haghdoost AA, Kheiry Z, Dehnavieh R (2019). Analysis of Iran’s national medical education evolution and innovation plan using the Michelle and Scott’s model of policymaking. J Adv Med Educ Prof.

[20] Pourabbasi A, Haghdoost A, Akbari H, Kheiry Z, Dehnavieh R, Noorihekmat S (2017). packages for reform and innovation in medical education in Islamic Republic of Iran; a conceptual framework. Teb va tazkiye J Minist Health Med Educ.

[21] Du Plessis P (2014). The principal as an instructional leader: guiding schools to improve instruction. Educ Change.

[22] Nir Z (2012). Rethinking public school leaders’ preparation as grounds for developing a training model. Mngr Chall Contemp Soc.

[23] Scribner SMP, Crow GM, Lopez GR, Murtadha K (2011). Successful principals: a contested notion for superintendents and principals. J School Leadersh.

[24] Asare KB (2016). Understanding the Transformational Leadership Practices of Colleges of Education Principals: Northcentral University.

[25] Yukl G (2012). Effective leadership behavior: what we know and what questions need more attention. Acad Manag Perspect.

[26] Groves KS, LaRocca MA (2011). An empirical study of leader ethical values, transformational and transactional leadership, and follower attitudes toward corporate social responsibility. J Bus Ethics.

[27] Diggs TR (2016). Exceptional leadership in exceptional times: perspective and ideologies of special education directors in southern California: Pepperdine University.

[28] Baldwina A, Millsb J, Birksc M, Buddenc L (2017). Reconciling professional identity: a grounded theory of nurse academics'role modelling for undergraduate students. Nurse Educ Today.

[29] Holloway I, Galvin K. Qualitative Research in Nursing and Healthcare, 4th Edition. Wiley-Blackwell; 2016.

[30] Starks H, Trinidad SB (2007). Choose your method: a comparison of phenomenology, discourse analysis, and grounded theory. Qual Health Res.

[31] Corbin J, Strauss A (2015). Basics of Qualitative Research :Techniques and Procedures for Developing Grounded Theory.

[32] Tong A, Sainsbury P, Craig J (2007). Consolidated criteria for reporting qualitative research (COREQ): a 32-item checklist for interviews and focus groups. Int J Qual Health Care.

[33] Corbin J, Strauss A (2008). Basics of qualitative research: Techniques and procedures for developing grounded theory: sage publication.

[34] Padgett DK (2016). Qualitative methods in social work research.

[35] Hsieh H, Shannon S (2005). Three approaches to qualitative content analysis. Qual Health Res.

[36] Hauserman CP, Stick SL (2013). The leadership teachers want from principals: transformational. Can J Educ.

[37] Ishikawa J (2012). Transformational leadership and gatekeeping leadership: the roles of norm for maintaining consensus and shared leadership in team performance. Asia Pac J Manag.

[38] Puaca G. Academic Leadership and Governance of Professional Autonomy in Swedish Higher Education, Scandinavian Journal of Educational Research. 2021;65(5):819-30. 10.1080/00313831.2020.1755359.

[39] Eddy PL (2013). Developing leaders: The role of competencies in rural community colleges. Community Coll Rev.

[40] Bakhsh K, Saadi AM, Rassol S (2014). What matters most? Determinants of administrative effectiveness. Am J Educ Res.

[41] Sahu AR, Shrivastava RR, Shrivastava RL (2013). Critical success factors for sustainable improvement in technical education excellence. The TQM journal.

[42] Gambhir V, Wadhwa NC, Grover S (2016). Quality concerns in technical education in India: a quantifiable quality enabled model. Qual Assur Educ.

[43] Dasgupta S. Reforms in medical education: optimizing quantity and quality. Indian J Public Health. 2014;58(1):1-4. 10.4103/0019-557X.128148. 10.4103/0019-557X.128148 24748349

[44] Boswell RA, Imroz SM (2013). The AACC leadership competencies: Pennsylvania's views and experiences. Community Coll J Res Pract.

[45] Keshavarzi MH, Soltani Arabshahi SK, Gharrahee B, Sohrabi Z, Mardani-Hamooleh M (2019). Exploration of faculty members’ perceptions about virtual education challenges in medical sciences: a qualitative study. J Adv Med Educ Prof.

[46] Meum TT, Koch TB, Briseid HS, Vabo GL, Rabben J (2021). Perceptions of digital technology in nursing education: a qualitative study. Nurse Educ Pract.

[47] Lai E (2014). Principal leadership practices in exploiting situated possibilities to build teacher capacity for change. Asia Pac Educ Rev.

[48] Srisaen K, Somprach K, Sombatteera S, Srisomjak S, Thana A (2014). Leadership practices of secondary school principals: A cross-national comparison of Thailand U.S. principals. Proc Soc Behav Sci.

